# Growth and activity of ANME clades with different sulfate and sulfide concentrations in the presence of methane

**DOI:** 10.3389/fmicb.2015.00988

**Published:** 2015-09-22

**Authors:** Peer H. A. Timmers, H. C. A. Widjaja-Greefkes, Javier Ramiro-Garcia, Caroline M. Plugge, Alfons J. M. Stams

**Affiliations:** ^1^Laboratory of Microbiology, Wageningen UniversityWageningen, Netherlands; ^2^Laboratory of Systems and Synthetic Biology, Wageningen UniversityWageningen, Netherlands; ^3^European Centre of Excellence for Sustainable Water TechnologyWetsus, Leeuwarden, Netherlands; ^4^Centre of Biological Engineering, University of MinhoBraga, Portugal

**Keywords:** methane oxidation, ANME, AOM, SR, sulfate reduction, niche separation

## Abstract

Extensive geochemical data showed that significant methane oxidation activity exists in marine sediments. The organisms responsible for this activity are anaerobic methane-oxidizing archaea (ANME) that occur in consortia with sulfate-reducing bacteria. A distinct zonation of different clades of ANME (ANME-1, ANME-2a/b, and ANME-2c) exists in marine sediments, which could be related to the localized concentrations of methane, sulfate, and sulfide. In order to test this hypothesis we performed long-term incubation of marine sediments under defined conditions with methane as a headspace gas: low or high sulfate (±4 and ±21 mM, respectively) in combination with low or high sulfide (±0.1 and ±4 mM, respectively) concentrations. Control incubations were also performed, with only methane, high sulfate, or high sulfide. Methane oxidation was monitored and growth of subtypes ANME-1, ANME-2a/b, and ANME-2c assessed using qPCR analysis. A preliminary archaeal community analysis was performed to gain insight into the ecological and taxonomic diversity. Almost all of the incubations with methane had methane oxidation activity, with the exception of the incubations with combined low sulfate and high sulfide concentrations. Sulfide inhibition occurred only with low sulfate concentrations, which could be due to the lower Gibbs free energy available as well as sulfide toxicity. ANME-2a/b appears to mainly grow in incubations which had high sulfate levels and methane oxidation activity, whereas ANME-1 did not show this distinction. ANME-2c only grew in incubations with only sulfate addition. These findings are consistent with previously published *in situ* profiling analysis of ANME subclusters in different marine sediments. Interestingly, since all ANME subtypes also grew in incubations with only methane or sulfate addition, ANME may also be able to perform anaerobic methane oxidation under substrate limited conditions or alternatively perform additional metabolic processes.

## Introduction

Anaerobic oxidation of methane (AOM) coupled to sulfate reduction (SR) has been found to occur in a wide range of marine sediments. The process is presumably performed by anaerobic methanotrophic archaea (ANME) in association with sulfate-reducing bacteria (SRB) belonging to the *Deltaproteobacteria* (Boetius et al., 2000). Recently, evidence has emerged that suggests ANME archaea can perform both AOM and SR to elemental sulfur (Milucka et al., 2012). The marine ANME clades presumed to be involved in AOM that have been described to date include ANME-1, ANME-2, and ANME-3. The ANME-2 clade has been further refined into subclusters a/b (previously considered to be two separate groupings) and subscluster c. The ANME-2 clade is closely related to cultivated members of the *Methanosarcinales*, the ANME-1 clade is related to *Methanomicrobiales* and *Methanosarcinales* (Hinrichs et al., 1999) and the ANME-3 clade is most related *Methanococcoides* spp. (Knittel et al., 2005). Due to this divergent taxonomy, it is expected that distinct ecological and physiological niches exist between the different ANME clades. The ANME clades 1 and 2 have been found in many different environments, whereas ANME-3 has been mainly found in mud volcanoes and some seep sediments (Niemann et al., 2006; Lösekann et al., 2007; Knittel and Boetius, 2009). Different ANME types do occur in the same marine environment, but show distinct zone formation in microbial mats or sediment cores. For instance, ANME-2 dominated surface layers of Hydrate Ridge sediments whereas ANME-1 was detected in deeper sediment layers with decreased sulfate and increased sulfide concentrations (Knittel et al., 2005). In another study, the ANME-2a/b was shown to be more predominant at low methane and low sulfide levels, with ANME-2c dominance occurring in deeper sediment layers closer to gas hydrates where the methane flux and sulfide concentration were relatively high (Roalkvam et al., 2011). Others have also observed an ecological transition of ANME-2a/b to ANME-2c and/or ANME-1 with increasing sediment depth (Orphan et al., 2004; Orcutt et al., 2005; Nunoura et al., 2006; Pachiadaki et al., 2011; Yanagawa et al., 2011; Roalkvam et al., 2012). As deduced from the data of Roalkvam et al. (2012), it is likely that a concentration below 5 mM sulfate has resulted in a shift from ANME-2a/b to ANME-1 and ANME-2c. These observations imply that there are distinct parameters that determine the distinctive ecological niches of different ANME subtypes. Direct characterization of the impact of these parameters on ANME subtypes would generate a deeper systematic understanding of the microbial ecology and physiology of AOM in marine sediments. Since many other uncontrolled factors are prevalent *in situ*, however, it is difficult to directly determine which factors actually influence ANME subtype presence, activity and growth.

We report here on the use of batch incubations to directly investigate AOM activity and growth of ANME subtypes under defined and controlled sulfate and sulfide concentrations in presence of methane. The experimental approach used slurries for inoculating the batch incubations. The slurries were prepared from Eckernförde bay sediment, which is an AOM mediating sediment known to contain ANME-1, ANME-2a/b, and ANME-2c subtypes. Incubation of the slurries was then performed using methane-oxidizing conditions with low sulfate (±4 mM) or high sulfate (±21 mM) concentrations in combination with low sulfide (±0.1–0.4 mM) or high sulfide (±3–4 mM) concentrations, respectively. Control incubations were also performed where only methane, high sulfate or high sulfide was added. Growth of ANME subtypes after 344 days was assessed by qPCR and compared to baseline values at the start of the incubations. In incubations which contained methane as headspace, after 540 days of incubation ^13^C-labeled CH_4_ was added to enable measurement of methane oxidation activity to be made until 947 days of incubation. Archaeal community analysis was performed on selected samples to observe differences in the ecological and taxonomic diversity of the different incubations.

## Materials and methods

### Origin of the inoculum

Samples were taken at Eckernförde Bay (Baltic Sea) at station B (water depth 28 m; position 54°31′15 N, 10°01′28 E) during a cruise of the German research vessel *Littorina* in June 2005. This sampling site has been described by Treude et al. (2005b). Sediment samples were taken with a small multicore sampler based on the construction described previously (Barnett et al., 1984). The cores had a length of 50 cm and reached 30–40 cm into the sediment bed. Immediately after sampling, the content of the cores was mixed in a large bottle, which was made anoxic by replacing the headspace by anoxic artificial seawater. Back in the laboratory, the headspace was replaced by CH_4_ (0.15 MPa) and the bottle was kept at 4°C in the dark.

### Media composition

The basal artificial marine medium used was prepared as described previously (Meulepas et al., 2009a). The mineral media did not contain any carbon source and no possible electron acceptors. The media were boiled, cooled down under a nitrogen (N_2_) flow and transferred into stock bottles. The headspace gas was exchanged 10 cycles with N_2_, with an end pressure of 1.5 bar N_2_ until use. The final pH of the media was 7.2. The phosphate provided buffering capacity to maintain a neutral pH-value.

### Experimental set-up

**Figure 1:**
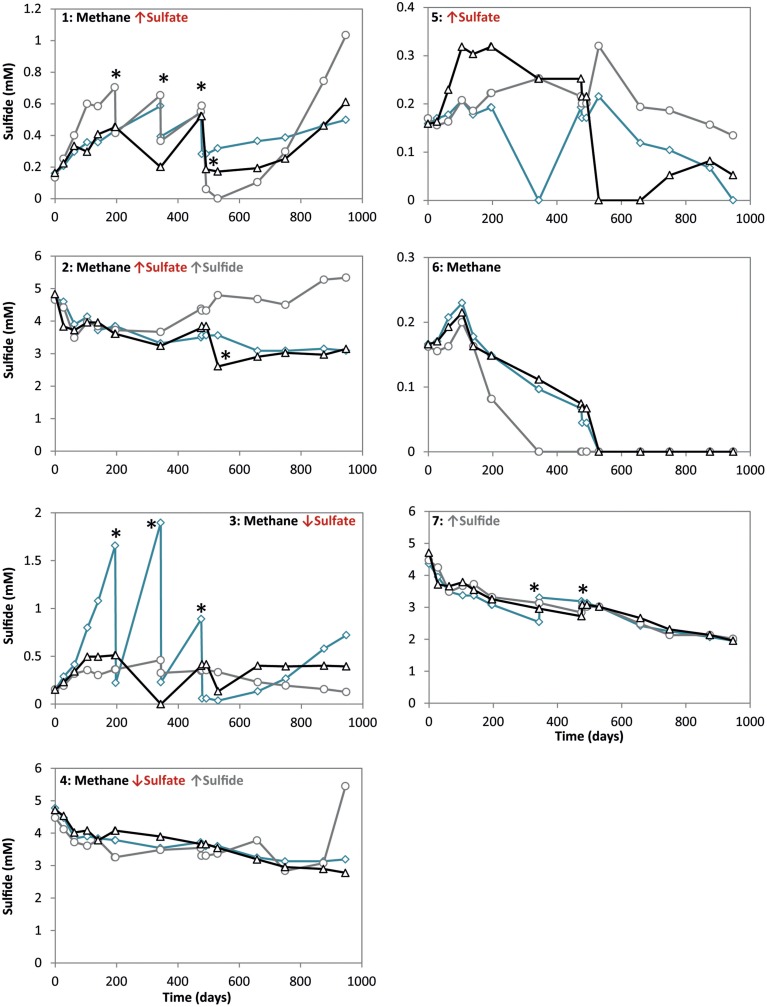
**Sulfide concentrations during 947 days of incubation in all conditions**. Arrows indicate either high (↑) or low (↓) sulfate and sulfide concentrations. The time points where either ferrous sulfate or sodium sulfate was added are indicated by an asterisk. Different lines represent triplicate incubations (A, blue diamonds; B, grey circles; C, black triangles).

**Figure 2:**
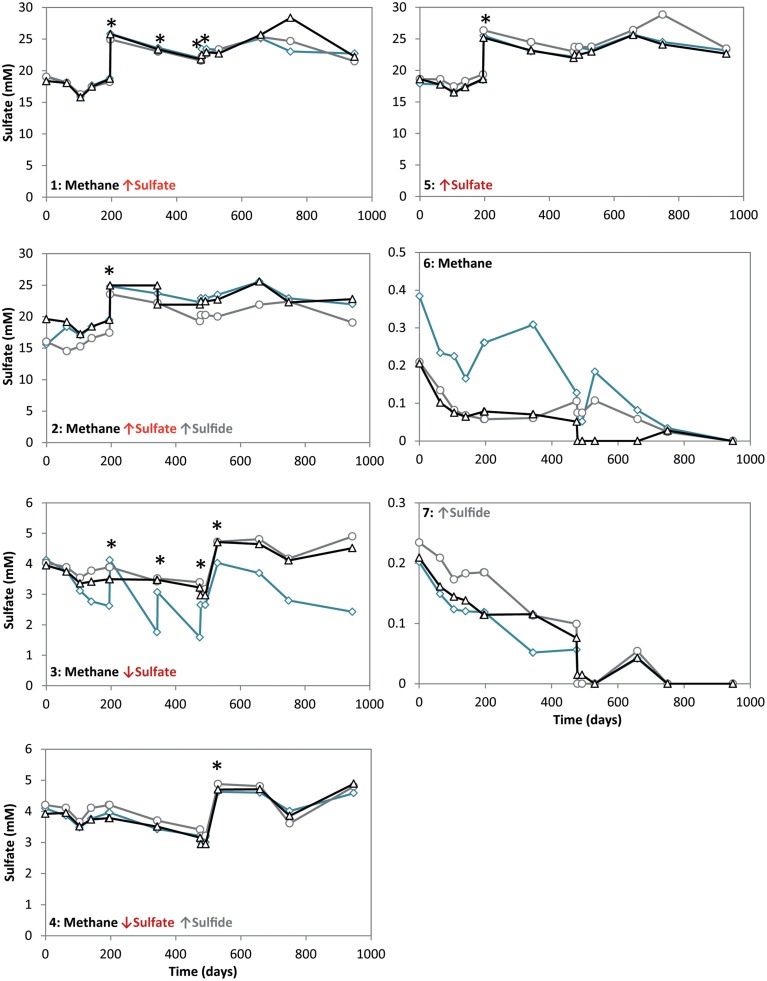
**Sulfate concentrations during 947 days of incubation in all conditions**. Arrows indicate either high (↑) or low (↓) sulfate and sulfide concentrations. The time points where either ferrous sulfate or sodium sulfate was added are indicated by an asterisk. Different lines represent triplicate incubations (A, blue diamonds; B, grey circles; C, black triangles).

For every condition, 30 ml Eckernförde bay sediment was incubated in triplicates with 90 ml of artificial marine medium in 244 ml serum bottles closed with butyl rubber stoppers and aluminum caps. Before inoculation, the headspace gas was exchanged 10 cycles with N_2_, with an end pressure of 1.8 bar N_2_ when no methane was added. When methane was added, the headspace gas was exchanged 10 cycles with 99.999% CH_4_ (Linde AG, Munich, Germany), with an end pressure of 1.8 bar CH_4_. Sulfide was then added to the artificial medium before inoculation to avoid toxicity effect of the concentrated sulfide stock solution. After sulfide addition, the pH was adjusted to 7.5 and then the bottles were inoculated. Serum bottles were horizontally incubated in the dark at 15°C without shaking. During incubation, sulfide, sulfate, and methane concentrations were monitored. Sulfide and sulfate concentrations for low and high conditions were kept at a constant concentration (Table 1). When sulfide concentrations were too high, a calculated amount of FeCl_2_ was added to precipitate excess sulfide. When sulfate concentrations were too low, Na_2_SO_4_ was added to obtain the desired concentration again. When sulfide concentrations were too high and sulfate concentrations were too low, FeSO_4_ was added to precipitate sulfide and to replenish sulfate (Figures 1, 2). After 540 days of incubation of the bottles where methane was added to the headspace, 99.999% CH_4_ was added to an end pressure of 1.6 bar. Then, 99.99% ^13^CH_4_ (Campro Scientific, Veenendaal, The Netherlands)was added to a final pressure of 1.8 bar. Sulfide and sulfate concentrations were determined and adjusted afterwards when necessary.

**Table 1:** **Experimental set-up and calculated Gibbs free energy changes**.

	**Condition**	**Sulfate (mM)**	**Sulfide (mM)**	**CH_4_(mM)**	**Total CO_2_ (mM)**	**Δ_*r*_G^′^**
Methane oxidizing	1: CH_4_ ↑ SO^2−^_4_	21.6	0.4	1.31	3.8	−28.1
	2: CH_4_ ↑ SO^2−^_4_↑ S^2−^	21.1	3.8	1.31	6.9	−21.1
	3: CH_4_ ↓ SO^2−^_4_	3.6	0.4	1.31	4.4	−23.4
	4: CH_4_ ↓ SO^2−^_4_↑ S^2−^	4.0	3.6	1.31	6.4	−17.4
Non-methane oxidizing	5: ↑ SO^2−^_4_	21.6	0.2	–	3.2	–
	6: CH_4_	0.1	0.1	1.31	2.7	−19.2
	7: ↑ S^2−^	0.4	3.0	–	4.4	–

*All conditions are performed in triplicate slurry incubations, sulfate, sulfide, and CO_2_ concentrations represent average concentrations of triplicates during 942 days of incubation (see Figures 1, 2 for detailed concentrations over time). Methane concentrations are theoretical maximum dissolved seawater concentrations (Yamamoto et al., 1976)*.

### Analytical measurements

Sulfate was analyzed by an Ion Chromatography system equipped with an Ionpac AS9-SC column and an ED 40 electrochemical detector (Dionex, Sunnyvale, CA). The system was operated at a column temperature of 35°C, and a flow rate of 1.2 ml min^−1^. Eluent consisted of a carbonate/bicarbonate solution (1.8 and 1.7 mM, respectively) in deionized water.

Headspace gas composition was measured on a gas chromatograph-mass spectrometer (GC-MS) composed of a Trace GC Ultra (Thermo Fisher Scientific, Waltham, MA) equipped with a Rt-QPLOT column (Restek, Bellefonte, PA), and a DSQ MS (Thermo Fisher Scientific, Waltham, MA). Helium was used as a carrier gas with a flow rate of 120 ml min^−1^ and a split ratio of 60. The inlet temperature was 80°C, the column temperature was set at 40°C and the ion source temperature at 200°C. The fractions of ^13^CO_2_, ^13^CH_4_, and ^12^CH_4_ were derived from the mass spectrum according to Shigematsu et al. (2004). Validation of the method was done using standards with known mixture of ^13^CO_2_, ^12^CO_2_, ^13^CH_4_, and ^12^CH_4_. For quantification of total CO_2_ and CH_4_, a compact GC (Global Analyser Solutions, Breda, The Netherlands) was used which contained a Carboxen 1010 pre-column, followed by two lines: a Molsieve 5A column (pressure: 200 kPa, split flow: 20 ml min^−1^, oven temperature: 80°C, and a PDD detector at 110°C) and a RT-Q-bond column (pressure: 150 kPa, split flow: 10 ml min^−1^. The concentrations of total CO_2_, total CH_4_, ^13^CO_2_, ^12^CH_4_ (produced during methanogenesis in incubations with ^13^CH_4_) and ^13^CH_4_ were calculated as described previously (Timmers et al., 2015).

Sulfide concentration was measured with the methylene-blue colorimetric method. Samples were directly diluted in 1:1 (v/v) in a 5% (w/v) zinc acetate solution to bind all sulfide. Deionized water was added to a volume of 4.45 ml, then 500 μl of reagent A (2 g l^−1^ dimethylparaphenylenediamine and 200 ml l^−1^ H_2_SO_4_) and 50 μl of reagent B (1 g l^−1^ Fe((NH_4_)(SO_4_))_2_. 12 H_2_O and 0.2 ml l^−1^ H_2_SO_4_) were added concurrently and mixed immediately. After 10 min, samples were measured with a Spectroquant Multy colorimeter (Merck Millipore, Darmstadt, Germany) at 660 nm.

The pressure of the serum vials was determined using a portable membrane pressure unit GMH 3150 (Greisinger Electronic GmbH, Regenstauf, Germany). The pH was measured using a solid gel epoxy electrode (Qis, Oosterhout, The Netherlands).

### DNA extraction

Genomic DNA was extracted from all triplicate incubations at the beginning of the experiment and after 344 days. Samples of 2 ml were taken at every time point and DNA was extracted using the Fast DNA Kit for Soil (MP Biomedicals, Solon, OH) according to the manufacturer's protocol with two 45-s beat beating steps using a Fastprep Instrument (MP Biomedicals). Afterwards, DNA was purified and concentrated using the DNA Clean and Concentrator kit (Zymo Research Corporation, Irvine, CA). DNA concentrations were determined with the Qubit 2.0 fluorometer (Thermo Fisher Scientific).

### Quantitative real-time PCR

Extracted DNA from the incubations at two time points (0 days and 344 days of incubation) was used for qPCR analysis as described previously (Timmers et al., 2015). Amplifications were done in triplicate in a BioRad CFX96™ system (Bio-Rad Laboratories, Hercules, CA) in a final volume of 25 μl using iTaq Universal SYBR Green Supermix (Bio-Rad Laboratories), 5 ng of template DNA and primers with optimal concentrations and annealing temperatures for highest efficiency and specificity (Supplementary Table 1), all according to the manufacturer's recommendations. Triplicate standard curves were obtained with 10-fold serial dilutions ranged from 2 × 10^5^ to 2 × 10^−2^ copies per μl of plasmids containing archaeal 16S rRNA gene inserts of ANME-1, ANME-2a/b and ANME-2c. The efficiency of the reactions was up to 100% and the R^2^ of the standard curves were up to 0.999. All used primers were extensively tested for specificity with cloned archaeal inserts of ANME-1, ANME-2a/b, ANME-2c, *Methanococcoides* and *Methanosarcinales* and genomic DNA of *Methanosarcina mazei* TMA (DSM-9195) and *Desulfovibrio sp*. G11 (DSM-7057).

For ANME-2a/b and ANME-1, PCR conditions consisted of a pre-denaturing step for 5 min at 95°C, followed by five touch-down cycles of 95°C for 30 s, annealing at 65°C for 30 s with a decrement per cycle to reach the optimized annealing temperature (temperatures are shown in Supplementary Table 1), and extension at 72°C (times are shown in Supplementary Table 1). This was followed by 40 cycles of denaturing at 95°C for 20 s for ANME-1 and 15 s for ANME-2a/b, and 30 s of annealing and extension at 72°C. For ANME-2c, PCR conditions consisted of a pre-denaturing step for 5 min at 95°C, followed by five cycles of 95°C for 30 s, annealing at 60°C for 30 s, and extension at 72°C for 40 s. This was followed by 40 cycles of denaturing at 95°C for 15 s, 30 s of annealing at 60°C and extension for 40 s at 72°C. PCR products were checked for specificity by a melting curve analysis (72–95°C) after each amplification step and gel electrophoresis. Quantification of specific archaeal groups was expressed as total 16S rRNA gene copies present per gram wet weight.

### Archaeal community analysis

Extracted DNA from selected samples (1C: CH_4_ ↑ SO^2−^_4_, 5A: ↑ SO^2−^_4_, 6B: methane only, 7A: sulfide only) at 344 days of incubation was used for archaeal community analysis. First amplification of archaeal 16S rRNA gene fragments was done using primers 518F (5′-CAGCMGCCGCGGTAA-3′) (Wang and Qian, 2009) and 905R (5′-CCCGCCAATTCCTTTAAGTTTC-3′) (Kvist et al., 2007). PCR amplification was performed in a total volume of 50 μl containing 500 nM of each forward and reverse primer (Biolegio BV), 1 unit of Phusion DNA polymerase (Thermo Scientific), 10 μl of HF-buffer, 200 μM dNTP mix, made to a total volume of 50 μl with nuclease free sterile water. The PCR program was as follows: denaturing at 98°C for 30 s, followed by 25 cycles of denaturing at 98°C for 10 s, annealing at 60°C for 20 s, extension at 72°C for 20 s, followed by a final extension step at 72°C for 10 min. A second amplification was performed to extend 8 nt barcodes to the generated amplicons, as used previously (Hamady et al., 2008). Barcoded amplification was performed in a total volume of 100 μl containing 5 μl of the first PCR product, 500 nM of each forward and reverse primer (Biolegio BV), 2 units of Phusion DNA polymerase (Thermo Scientific), 20 μl of HF-buffer, 200 μM dNTP mix, made to a total volume of 100 μl with nuclease free sterile water. The PCR program was as follows: denaturing at 98°C for 30 s, followed by five cycles of denaturing at 98°C for 10 s, annealing at 52°C for 20 s, extension at 72°C for 20 s, followed by a final extension at 72°C for 10 min. Barcoded PCR products were cleaned using the HighPrep PCR clean-up system (MagBio Genomics Inc., Gaithersburg, MD). DNA concentrations were quantified using Qubit (Invitrogen, Bleiswijk, The Netherlands). Afterwards, barcoded samples were pooled in equimolar quantities, purified using the MagBio HighPrep PCR- 96 well protocol and then quantified using Qubit. Samples were submitted for MiSeq sequencing on the Illumina platform using sequencing by synthesis chemistry.

### Sequencing data analysis

For analysis of the 16S RNA gene sequencing data, an in-house pipeline was used (Ramiro-Garcia et al., unpublished). Shortly, paired-end libraries were filtered to contain only read pairs with perfectly matching primer and barcodes. Resulting reads were separated by sample using the barcode and operational taxonomic units (OTUs) were assigned using an open reference approach and a customized SILVA 16S rRNA reference database (Quast et al., 2013). Microbial composition plots were generated using a workflow based on Quantitative Insights Into Microbial Ecology (QIIME) v1.2 (Caporaso et al., 2010). The project was deposited to the SRA archive of the European Nucleotide Archive (ENA) with the study accession number PRJEB10324 (http://www.ebi.ac.uk/ena/data/view/PRJEB10324).

## Results

### qPCR and archaeal community analysis

**Figure 3:**
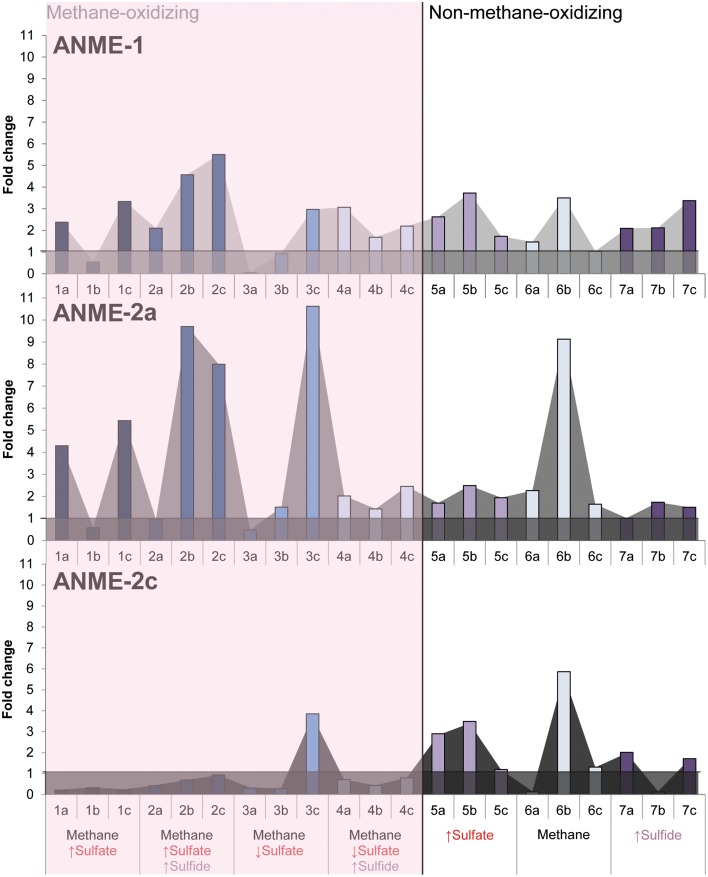
**The fold change of ANME-1, ANME-2a/b and ANME-2c copies ng DNA^−1^ g wet weight^−1^ after 344 days of incubation in all conditions**. Arrows indicate either high (↑) or low (↓) sulfate and sulfide concentrations. The horizontal lines at a fold change of 1 show fold changes that indicate growth.

Growth of ANME subclades was observed in all conditions (Supplementary Figures 1–3). The Eckernförde bay sediment inoculum contained mostly ANME-2a/b, few ANME-1 and even less ANME-2c copies. The fold increase of ANME-1, ANME-2a/b, and ANME-2c therefore gives more information on which subtype increased most in copy numbers during incubation. Figure 3 shows that at almost all methane-oxidizing conditions, the ANME-2a/b subtype had a higher fold increase than ANME-1 and ANME-2c, except where sulfate is low and sulfide is high. ANME-2a/b archaea apparently grew best when sulfate was supplied in high concentrations in presence of methane, with or without high levels of sulfide (Figure 3). The ANME-1 subtype also seemed to proliferate under methane-oxidizing conditions, but showed similar growth in all other conditions. ANME-2c seemed to grow only when sulfate but no methane was added. These results were confirmed by archaeal community analysis that showed higher abundance of ANME-2a/b in condition with methane as compared to conditions without methane (Supplementary Figure 5). ANME-1 seemed to be equally abundant in every condition sequenced, except for the condition with only sulfide where it showed a slightly higher relative abundance (Supplementary Figure 5).

### AOM activity

**Figure 4:**
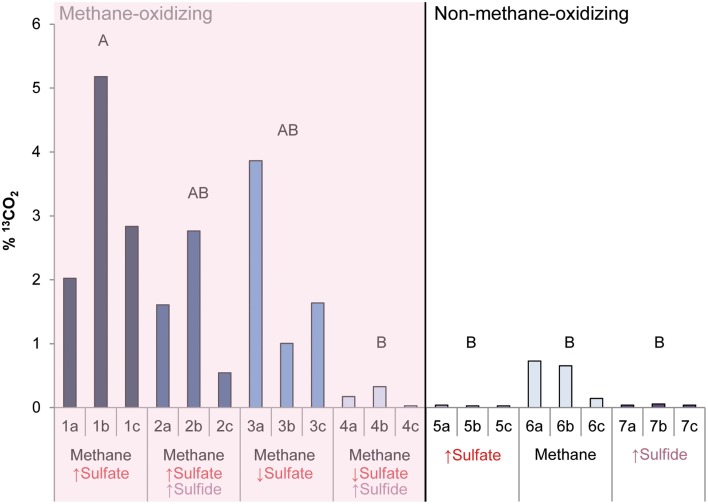
**The produced ^13^CO_2_ (%) after ^13^CH_4_ addition between 540 and 947 days of incubation in all conditions**. Arrows indicate either high (↑) or low (↓) sulfate and sulfide concentrations. Different letters represent significant difference (*t*-test with unequal variance, *p* < 0.05).

During the whole incubation period of 947 days, sulfate and sulfide concentrations were monitored and controlled at the desired level. In high sulfide incubations, sulfide concentrations were decreasing over time and sodium sulfide had to be added at some time points, where the condition with only sulfide showed highest sulfide decrease (Figure 1). In incubations with low sulfide concentrations, sulfide was produced only in presence of methane, and no sulfide production occurred in the absence of methane (Figure 1). Conditions with methane and high sulfate concentrations showed highest sulfate reduction and sulfide had to be precipitated with ferrous sulfate regularly, whereas with methane and low sulfate, sulfate reduction was lower with the exception of one of the triplicates (Figure 2). Although sulfate reduction was highest in condition with high sulfate concentrations, sulfate reduction did take place in all conditions, even where no sulfate was added; the endogenous sulfate of less than 0.4 mM was completely reduced (Figure 2). When ^13^C- labeled methane was added to the headspace at day 540 of methane-containing conditions, ^13^CO_2_ production was also monitored. Production of ^13^CO_2_ was apparent in all conditions, except where no ^13^CH_4_ was added (Figure 4). Only the combined addition of high sulfate with low sulfide showed significantly higher AOM rates relative to the non-methane-oxidizing conditions (*t*-test unequal variance, *p* < 0.05). When sulfate was high, there was no substantial difference between low sulfide and high sulfide addition. When sulfide was low, there was no substantial difference between high sulfate and low sulfate addition. However, there was a substantial difference when sulfate was high together with low sulfide and when sulfate was low together with high sulfide (*t*-test unequal variance, *p* < 0.05). Methane oxidation was associated with sulfide production in conditions with methane and sulfate when sulfide levels were low (Supplementary Figure 4). Almost no methane production was observed in conditions with “sulfate only” addition; 0.2 mM methane at 659 days of incubation in all triplicates and 0.9 mM in one of the triplicates at 947 days of incubation. Both methane peaks were not detectable upon further incubation. Triplicate incubations of the condition with “sulfide only” addition did show methane accumulation up to 3.7, 1.4, and 1.0 mM after 947 days.

For each experimental condition, Δ_*r*_G′ values were calculated (Table 1) according to the Δ_*r*_G°′of −21 kJ mol^−1^, assuming methane as gas molecule and products to be in the form of CO_2_ and HS^−^ (Thauer, 2011). For the calculation, we used the maximum dissolved methane concentration of 1.31 mM at a salinity of 30 and 20°C (Yamamoto et al., 1976), assuming the effect of 0.5–1 bar overpressure is negligible to increase solubility. The CO_2_, sulfide and sulfate concentrations used for the calculations are also given in Table 1. These concentrations were the average concentration during 947 days of incubation in triplicate incubations. Detailed concentrations over time are given in Figure 1 for sulfide and Figure 2 for sulfate. According to the calculations, the lowest Gibbs free energies (most negative) were associated with high sulfate and low sulfide concentrations, and the Gibbs free energy changes were smallest with low sulfate and high sulfide concentrations.

## Discussion

### Methane-oxidizing conditions

Growth of ANME-2a/b was apparent when methane was added and was highest with high sulfate concentrations, independent of the sulfide concentration. Only when sulfate was low together with high sulfide, was growth substantially less. This was confirmed by archaeal 16S rRNA gene sequencing. Methane oxidation was also substantially lower with low sulfate and high sulfide concentrations, compared to high sulfate and low sulfide concentrations. Moreover, no substantial difference in AOM rates between high and low sulfate addition in the presence of methane was observed. It was shown before with the same Eckernförde bay sediment enriched in ANME-2a/b that AOM rates only became affected when sulfate concentrations were below 2 mM. This indicates that the K_m_ for sulfate is lower than 2 mM (Meulepas et al., 2009b) and thus 5 mM sulfate should not result in lower rates of AOM. Therefore, the sulfate and sulfide concentrations seemed to have a combined effect in terms of determining growth and activity of ANME-2a/b.

The Δ_*r*_G′ of methane-oxidizing conditions are similar to reported ΔG′ values for non-seep environments with low methane concentrations (-18 to −25 kJ mol methane^−1^) (Caldwell et al., 2008; Thauer, 2011). These values were previously argued to be close to the minimum energy required to sustain life. The translocation of one proton over the membrane, the minimum biological energy quantum, was calculated to have a ΔG′ of around −19 kJ mol sulfate^−1^ for sulfate-reducing bacteria and −10.6 kJ mol methane^−1^ for methanogenic archaea (Hoehler et al., 2001). This corresponded to estimates of critical energy yields for ANME/SRB aggregates of −10 kJ mol methane^−1^ (Alperin and Hoehler, 2009), and activity at the calculated value of −10.6 kJ mol^−1^ methane was confirmed *in situ* (Nauhaus et al., 2002). Until now, it has been unclear if AOM activity under the least favorable conditions could still be associated with microbial growth. Conditions which were above the threshold of −19 kJ mol methane^−1^ in this study did show AOM activity and associated growth of ANME-2a/b, while other conditions showed substantially less activity and growth. This implies that below this threshold, ANME-2a/b growth was probably inhibited in our incubations. Since growth and activity of ANME-2a/b seems to be directly related to the theoretical available energy, this explains the sulfide dependency only at low sulfate concentrations. The low ΔG′ values of AOM in non-seep sediments as compared to seep sediments (−35 kJ mol methane^−1^) (Valentine and Reeburgh, 2000; Caldwell et al., 2008; Alperin and Hoehler, 2009; Wang et al., 2010; Thauer, 2011) are mainly due to the differences in the dissolved methane concentrations. Sulfate and sulfide concentrations can therefore have a larger effect on AOM rates in non-seep systems relative to seep systems as with similar sulfate and sulfide concentrations, the ΔG' of the reaction can still stay low enough for the reaction to occur in seep systems. This could explain the lack of sulfide inhibition in seep sediments that showed AOM activity under conditions where 10–15 mM sulfide was produced (Nauhaus et al., 2002; Valentine, 2002; Joye et al., 2004). However, sensitivity to sulfide toxicity may also differ between ANME species, as an AOM performing enrichment has also been reported to have complete inhibition at 2.5 mM of sulfide, despite AOM still being thermodynamically feasible (Meulepas et al., 2009a,b).

ANME-1 seemed to grow in almost every condition, and did not show the differential growth characteristics that ANME-2a/b did. On this basis, under methane-oxidizing conditions, it could be predicted that the ANME-2a/b subtype would outcompete ANME-1 in the presence of high amounts of sulfate (with either high or low amounts of sulfide) but not in the combined presence of low amounts of sulfate and high amounts of sulfide. This is generally consistent with the *in situ* observations of ANME-1 thriving in low-methane (Blumenberg et al., 2004; Elvert et al., 2005), methane-free (Bertram et al., 2013), sulfate-depleted environments (Yanagawa et al., 2011; Vigneron et al., 2013), and in environments with elevated sulfide levels (Knittel et al., 2005; Krüger et al., 2008; Biddle et al., 2012). ANME-2a/b thrive at low sulfide levels (Knittel et al., 2005; Roalkvam et al., 2011; Biddle et al., 2012), high sulfate concentrations (Rossel et al., 2011; Yanagawa et al., 2011) or both (Krüger et al., 2008). Higher sulfate concentrations generally occur close to the surface of sediments where ANME-2a/b are generally found to be dominant (Orphan et al., 2004; Orcutt et al., 2005; Nunoura et al., 2006; Pachiadaki et al., 2011; Roalkvam et al., 2012). Moreover, previous reactor studies with Eckernförde bay sediment where sulfate was kept high and sulfide was kept low were successful in obtaining high rates of AOM and enrichment of ANME-2a/b archaea (Meulepas et al., 2009a).

### Non-methane-oxidizing conditions

Under non-methane-oxidizing conditions where growth of ANME archaea was observed, it is possible that the ANME could still perform AOM or potentially another process. In the “methane only” condition, growth of all ANME types and methane oxidation was apparent. Archaeal community analysis also showed a higher abundance of ANME-2a/b as relative to “sulfate only” and “sulfide only” conditions. With the “methane only” condition, methane oxidation was probably partly coupled to sulfate reduction since the 0.2–0.4 mM sulfate that was present was completely reduced during incubation. It has been reported that AOM occurs even below 0.5 mM sulfate, but at lower rates than at higher sulfate concentrations (Wegener and Boetius, 2009; Meulepas et al., 2009b; Beal et al., 2011; Yoshinaga et al., 2014). However, it has also been reported that when labeled methane is used in incubations, methanogenesis can also produce labeled CO_2_ in a process called “trace methane oxidation” (Zehnder and Brock, 1979). Archaeal community analysis showed high abundance of *Methanococcoides* in the “methane only” condition, indicating that methanogenesis (and thus trace methane oxidation) and AOM did indeed co-occur.

In the “sulfate only” conditions, very little sulfide production was found, suggesting that endogenous substrates were an unimportant source for sulfate reduction. We did observe presence of methane at two time points (0.2 mM and 0.9 mM at day 659 and 947, respectively), indicating that methane was produced. In theory, this methane could have been oxidized since the 0.2 mM could not be detected at day 750. At 0.2 mM methane, the ΔG′ of the reaction already is −25.7 kJ, which is sufficiently negative for growth of ANME. However, since methane was not measurable at other time points, it is not clear if “sulfate only” conditions were favorable for AOM throughout the experiment.

In the condition with “sulfide only,” both sulfate reduction and methane production (3.7, 1.4, and 1.0 mM methane in biological triplicates after 947 days) took place, making AOM possible. However, at these conditions, the ΔG′ was a maximum of −13.1 kJ mol^−1^. Methane accumulated slowly throughout the experiment under this conditions, in contrast to the “sulfate only” condition. It is likely that this was due to the energetic yield being too low to allow significant AOM activity. In both “sulfate only” and “sulfide only” conditions, we did not find an abundance of methanogens (< 0.5% of reads belonging to *Methanococcoides* under both conditions, and < 0.2% of reads of *Methanobacteriaceae* in “sulfide only” condition) although methanogenesis did occur. In the “sulfide only” condition, ANME-1 grew and appeared to be relatively more abundant than in the other conditions analyzed by 16S rRNA gene sequencing. ANME-1 cells have been frequently observed to occur without a bacterial partner (Orphan et al., 2002, 2004; Blumenberg et al., 2004; Knittel et al., 2005) and it has been postulated that ANME-1 can either oxidize methane alone (Orphan et al., 2002; Pachiadaki et al., 2011; Maignien et al., 2013). Indications also exist that ANME-1 can perform methanogenesis (Lloyd et al., 2011) as was also found for ANME-2 (Bertram et al., 2013).

Yoshinaga et al. (2014) showed that when the ΔG′ of AOM decreases (from −35 to −20 kJ mol^−1^) due to sulfate depletion and sulfide accumulation, the AOM back flux becomes significant. As the AOM rates decrease, the AOM back flux increases, resulting in significant production of methane from CO_2_ (Yoshinaga et al., 2014). The observed ^13^C depletion below the sulfate-methane transition zone (SMTZ) in marine sediments that was previously thought to come from methanogenesis, may thus come from the back flux of AOM. This corresponds to reports of high AOM activities below the SMTZ in the methanogenic zone (Treude et al., 2005a; Parkes et al., 2007; Knab et al., 2009; Yoshioka et al., 2010). This back flux could have been occurring in the “methane only” and “sulfate only” conditions, which could also explain growth of ANME subtypes.

Insight into the diversity of potential metabolic properties of ANME archaea also derived from metagenomic and metaproteomic studies of ANME-1 and ANME-2a/b methanotrophs (Hallam et al., 2004; Meyerdierks et al., 2005, 2010; Stokke et al., 2012; Wang et al., 2014). For instance, ANME-2a/b seems to have the potential to metabolize acetate (Wang et al., 2014) and although the canonical dissimilatory sulfate reduction pathway was not present, potential alternative pathways for sulfate reduction have been postulated to exist in ANME-1 (Meyerdierks et al., 2005, 2010; Stokke et al., 2012). It was experimentally shown that ANME-2 archaea could reduce sulfate to elemental sulfur coupled to methane oxidation (Milucka et al., 2012). This indeed implied that ANME could use alternative pathways for sulfate reduction. Therefore, more research on the metabolic capabilities of ANME, especially sulfate reduction and methanogenesis, could also explain presence of ANME at unexpected sites where methane oxidation activity is not observed.

## Author contributions

PT designed the experiment and conducted most experimental work and wrote the article. HW performed the qPCR analysis and helped with analysis and interpretation of the work. JR performed community analysis and helped with analysis and interpretation of the work. CP and AS contributed with design of the experiment and interpretation of the data, revising the intellectual content and approved the final version to be published. All authors agreed to be accountable for all aspects of the work.

### Conflict of interest statement

The authors declare that the research was conducted in the absence of any commercial or financial relationships that could be construed as a potential conflict of interest.
