# Supplementary material for: Growth and activity of ANME clades with different sulfate and sulfide concentrations in the presence of methane
Source: Front Microbiol. 2015 Sep 22;6:988. doi: 10.3389/fmicb.2015.00988 (PMC4585129; doi:10.3389/fmicb.2015.00988)
Supplement: Supplementary file 1 [file SupplementaryData.PDF]

Supplementary Material

Growth and activity of ANME clades with different sulfate and sulfide concentrations in the presence of methane

Peer H.A. Timmers*, H.C. Aura Widjaja-Greefkes, Javier Ramiro-Garcia, Caroline M. Plugge and Alfons J.M. Stams

***Correspondence:** Corresponding Author: peer.timmers@wur.nl

1. Supplementary Figures and Tables

1.1. Supplementary Figures

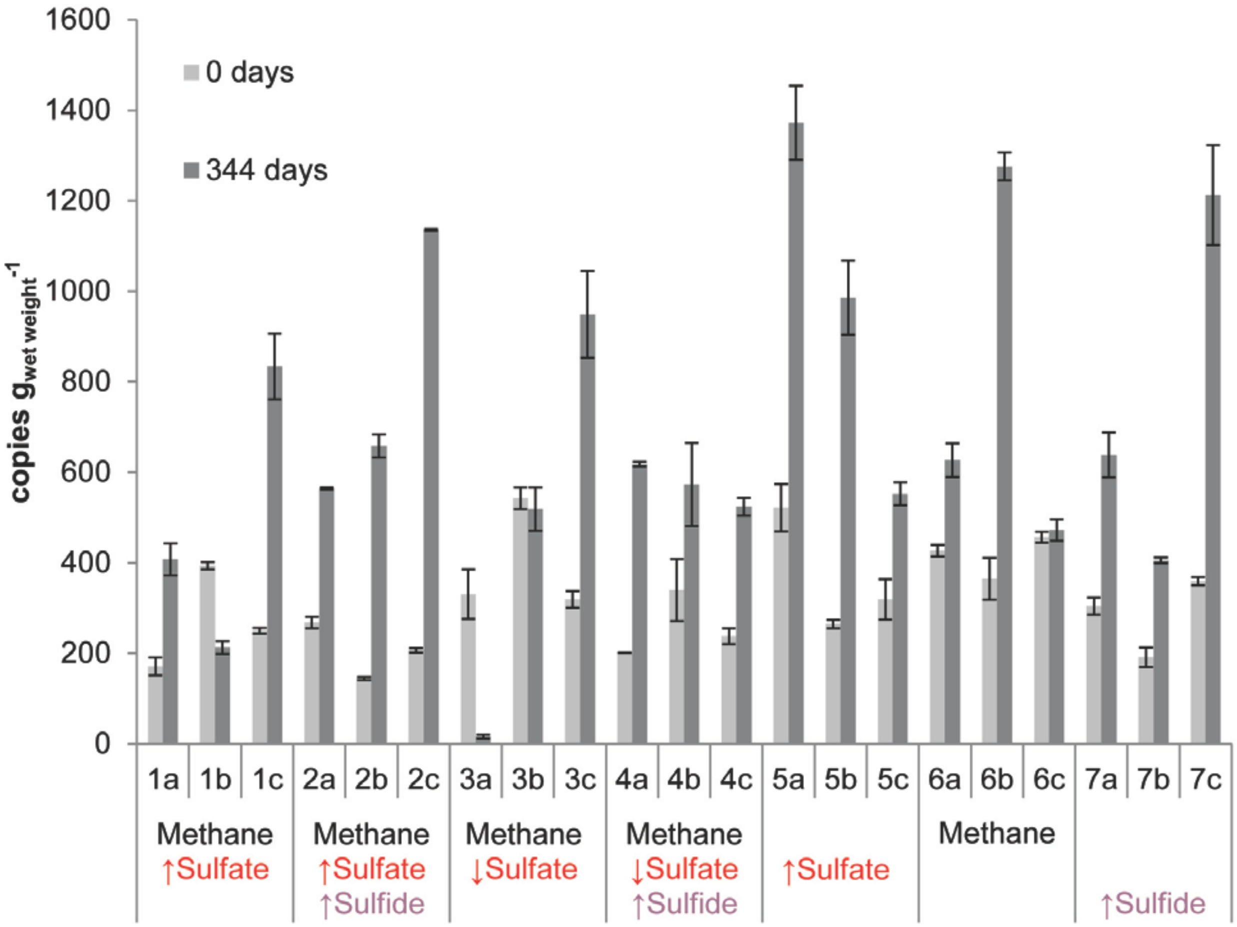

Supplementary Figure 1 qPCR results of specific primers for ANME-1 expressed as the absolute amount of ANME-1 (copies g wet weight^{-1}) at 0 days and at 344 days of incubation. Arrows at conditions on the x-axis indicate either high (↑) or low (↓) sulfate and sulfide concentrations.

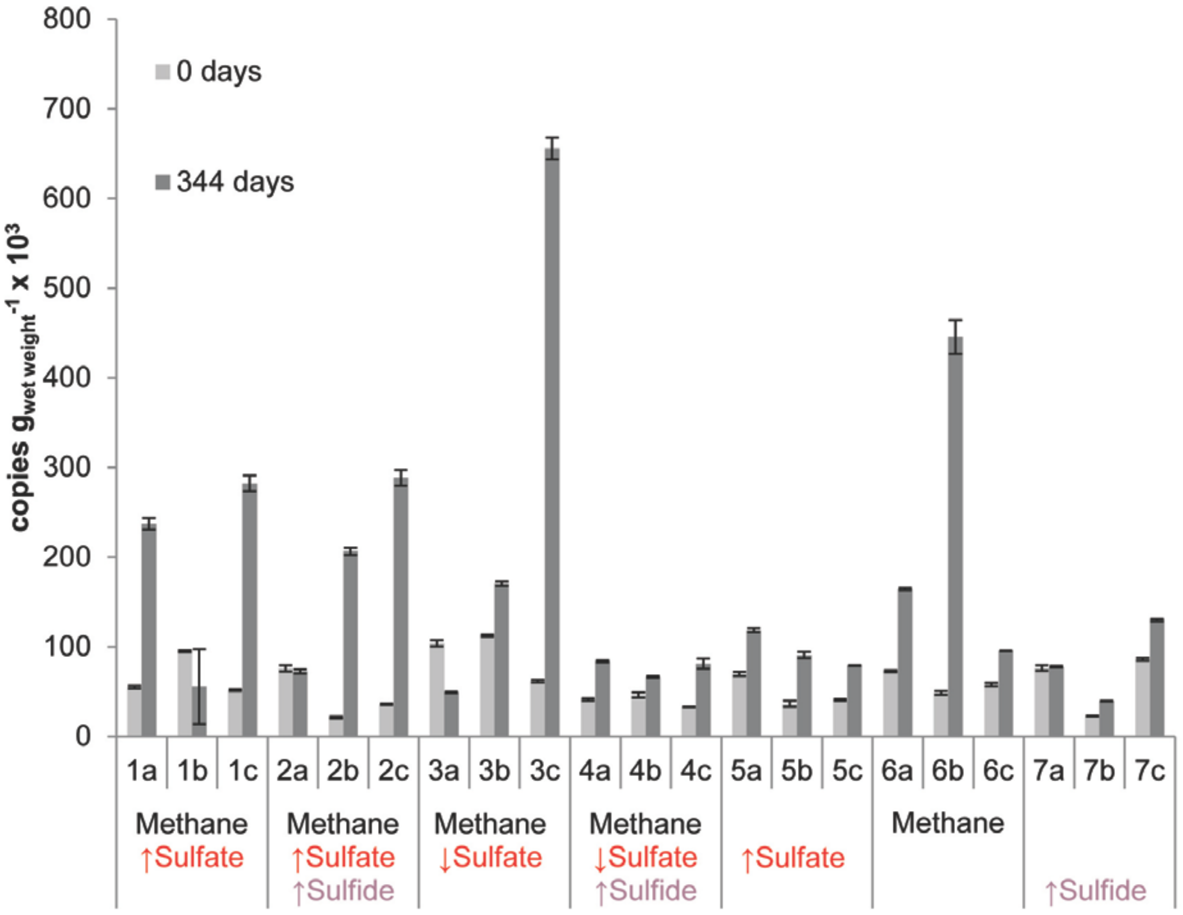

Supplementary Figure 2 qPCR results of specific primers for ANME-2a/b expressed as the absolute amount of ANME-2a/b (copies g wet weight^{-1}) at 0 days and at 344 days of incubation. Arrows at conditions on the x-axis indicate either high (↑) or low (↓) sulfate and sulfide concentrations.

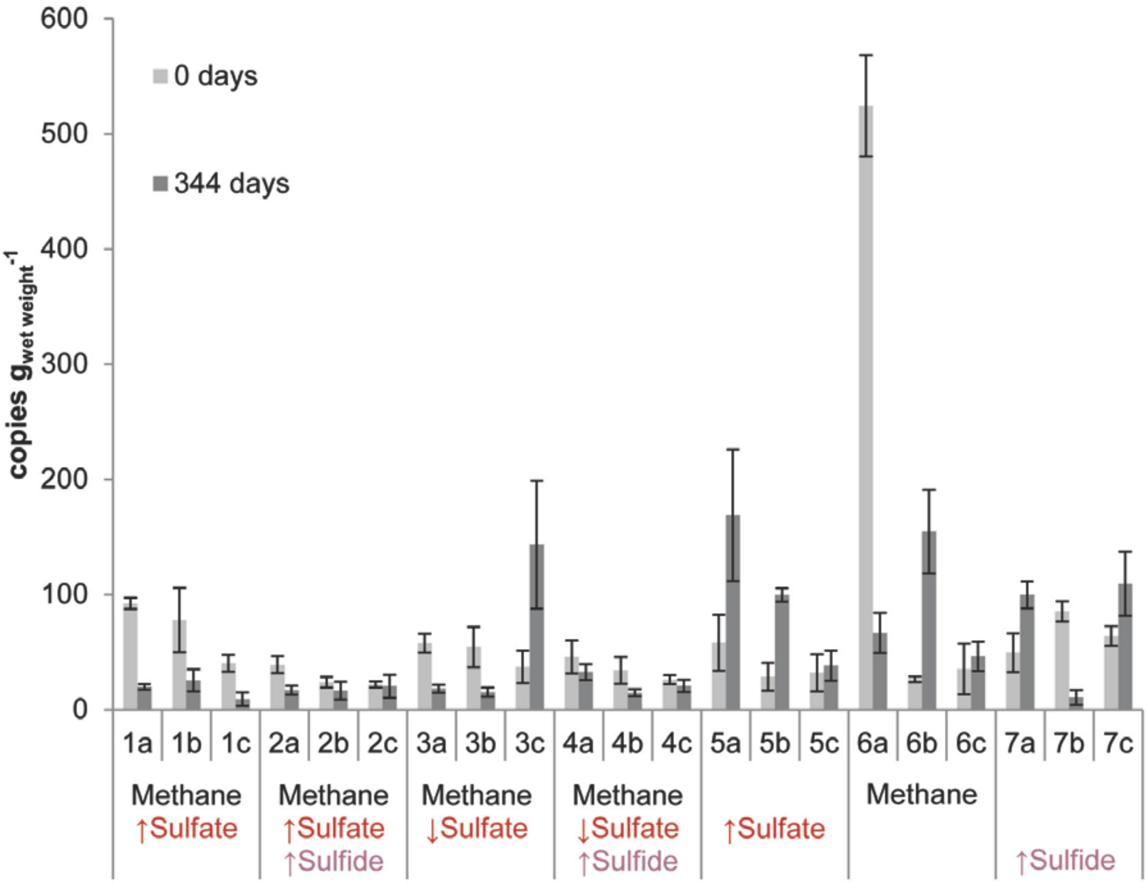

Supplementary Figure 3 qPCR results of specific primers for ANME-2c expressed as the absolute amount of ANME-2c (copies g wet weight^{-1}) at 0 days and at 344 days of incubation. Arrows at conditions on the x-axis indicate either high (↑) or low (↓) sulfate and sulfide concentrations.

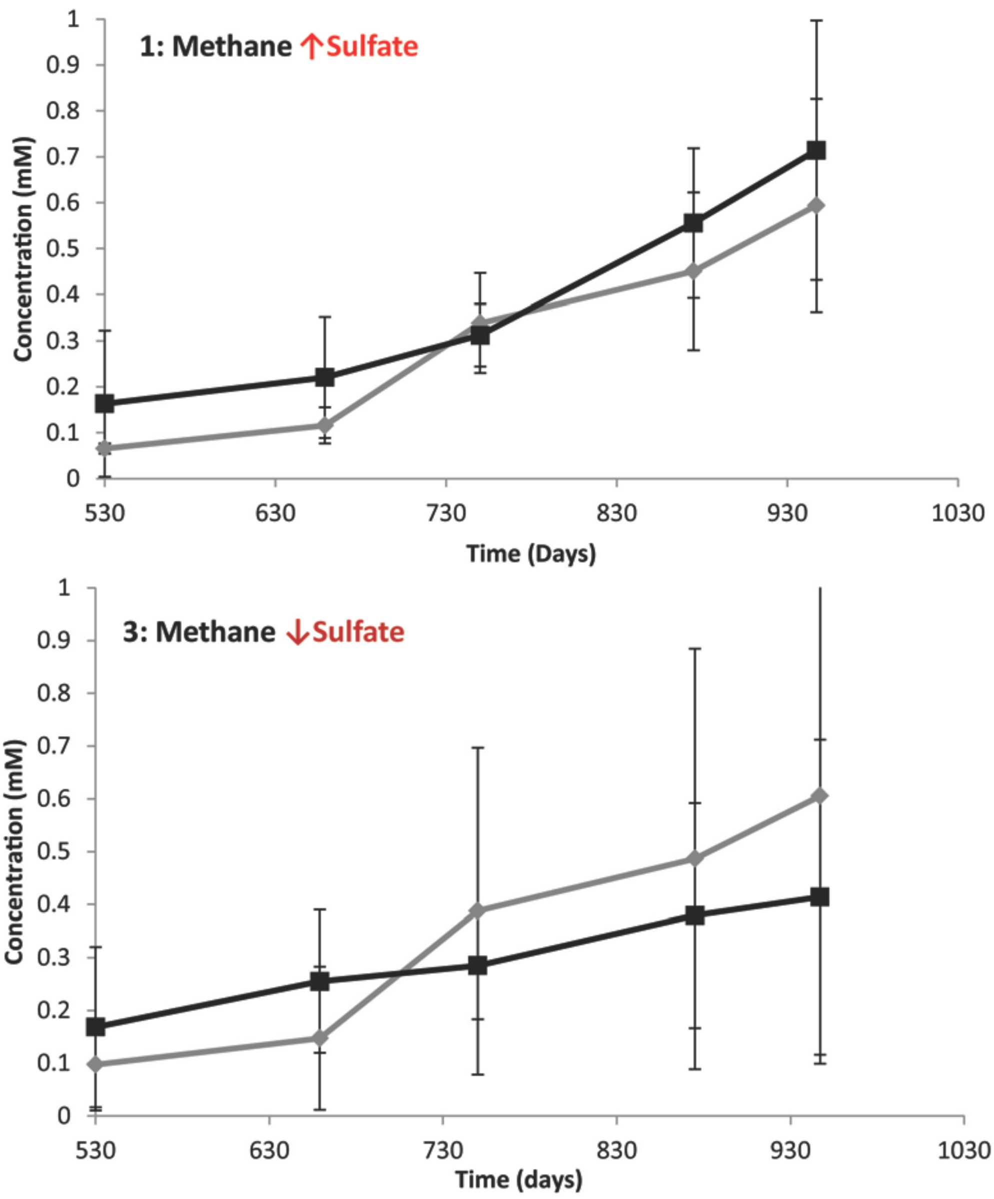

Supplementary Figure 4 Sulfide (squares, black lines) and $^{13}CO_2$ concentration (diamonds, grey lines) in mM in methane-oxidizing conditions 1 and 3 (arrows indicate either high (↑) or low (↓) sulfate and sulfide concentrations) incubated with $^{13}CH_4$ after 530 days of incubation. Standard deviations represent triplicate incubations.

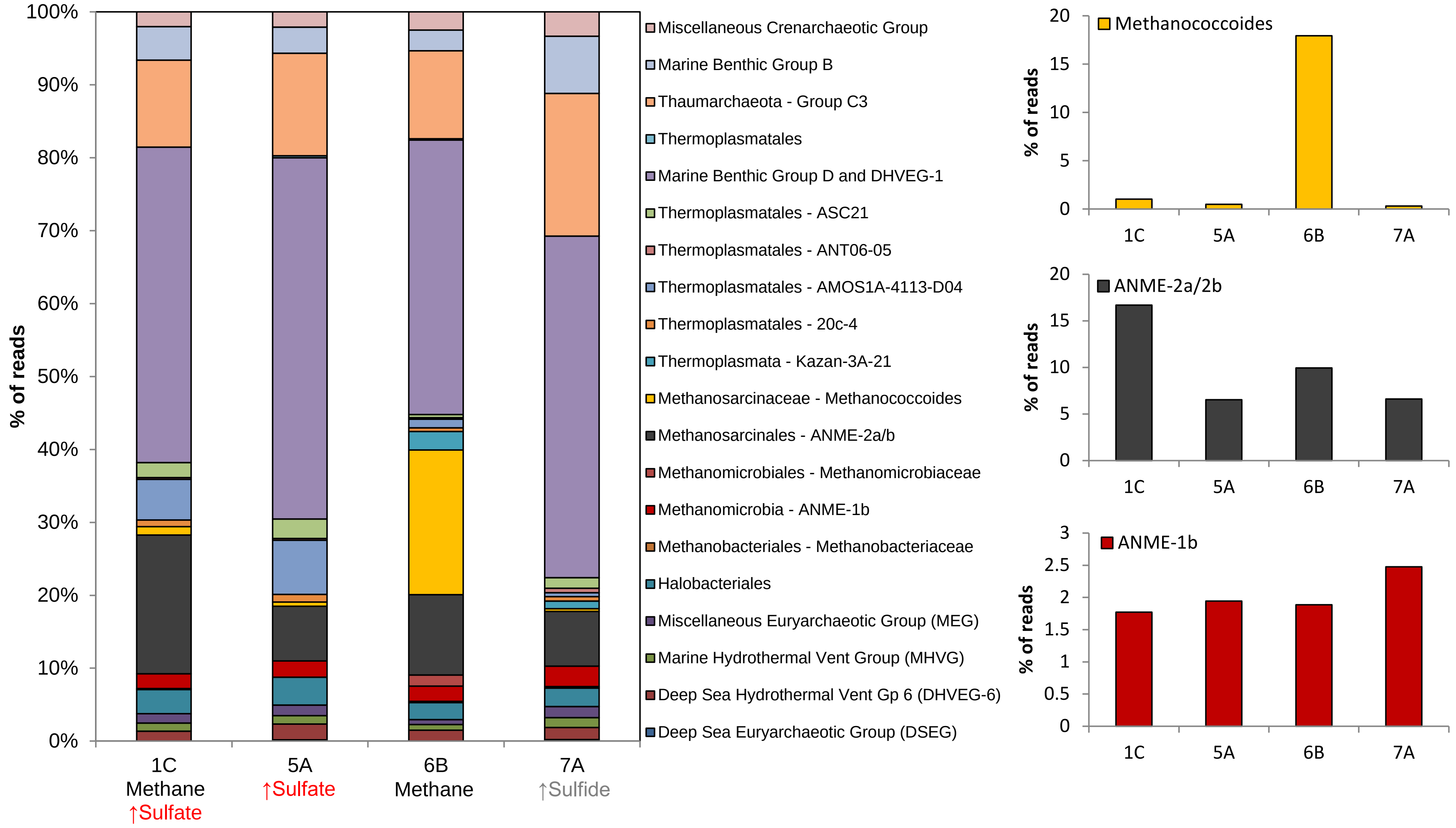

Supplementary Figure 5 Archaeal community analysis results of one of the triplicates of conditions with methane and high sulfate (1C), only sulfate (5A), only methane (6B) and only sulfide (7A) showing the relative amount of reads (%) of all sequences retrieved (left panel) and detailed view of ANME-2a/b, ANME-1 and *Methanococcoides* (right panel).

1.2. Supplementary Tables

Supplementary Table 1 Primers used for qPCR in this study with the corresponding annealing temperatures and concentrations.

Target	Primer name	Sequence (5'-3')	Annaeling temp (°C)	Extension time (s)	Amplicon size (bp)	Primer conc. (mM)	Ref.
ANME-1	ANME-1-395F*	AAC TCT GAG TGC CTC CTA	57.5	40	1039	1	(Miyashita et al 2009)
		AAC TCT GAG TGC CTC CAA					
		AAC TCT GAG TGC CCC CTA					
	ANME-1-1417R*	CCT CAC CTA AAC CCC ACT				1	
		CCT CAC CTA AAT CCC ACT					
ANME-2a/b	ANME2a-426F*	TGT TGG CTG TCC GGA TGA	60	40	833	1	(Miyashita et al 2009)
		TGT TGG CTG TCC AGA TGA					
		TGT TGG CTG TCC AGA TGG					
	ANME2a-1242R	AGG TGC CCA TTG TCC CAA				1	
ANME-2c	ANME-2cF	TCG TTT ACG GCT GGG ACT AC	60	40	221	1	(Vigneron et al 2013)
	ANME-2cR	TCC TCT GGG AAA TCT GGT TG				1	

*These primers are a mixture of each taxonomic group targeted primers at an equimolar amount, as described by Miyashita et al., 2009

References

Miyashita A, Mochimaru H, Kazama H, Ohashi A, Yamaguchi T, Nunoura T *et al* (2009). Development of 16S rRNA gene-targeted primers for detection of archaeal anaerobic methanotrophs (ANMEs). *FEMS Microbiol Letters* **297:** 31-37.

Vigneron A, Cruaud P, Pignet P, Caprais JC, Cambon-Bonavita MA, Godfroy A *et al* (2013). Archaeal and anaerobic methane oxidizer communities in the Sonora Margin cold seeps, Guaymas Basin (Gulf of California). *ISME J* **7:** 1595-1608.
